# Additive Production of a Material Based on an Acrylic Polymer with a Nanoscale Layer of Zno Nanorods Deposited Using a Direct Current Magnetron Discharge: Morphology, Photoconversion Properties, and Biosafety

**DOI:** 10.3390/ma14216586

**Published:** 2021-11-02

**Authors:** Dmitry E. Burmistrov, Denis V. Yanykin, Mark O. Paskhin, Egor V. Nagaev, Alexey D. Efimov, Andrey V. Kaziev, Dmitry G. Ageychenkov, Sergey V. Gudkov

**Affiliations:** 1 Prokhorov General Physics Institute of the Russian Academy of Sciences, 38 Vavilova St., 119991 Moscow, Russia; dmitriiburmistroff@gmail.com (D.E.B.); ya-d-ozh@rambler.ru (D.V.Y.); pashin.mark@mail.ru (M.O.P.); egorn97@mail.ru (E.V.N.); alefimov1997@gmail.com (A.D.E.); 2 Moscow Engineering Physics Institute, National Research Nuclear University MEPhI, Kashirskoe Sh. 31, 115409 Moscow, Russia; kaziev@plasma.mephi.ru (A.V.K.); dgageichenkov@mephi.ru (D.G.A.)

**Keywords:** ZnO, reactive magnetron sputtering, sputtering in an argon-oxygen mixture, photoconversion materials, agrophotonics, biocompatibility

## Abstract

On the basis of a direct current magnetron, a technology has been developed for producing nanoscale-oriented nanorods from zinc oxide on an acrylic polymer. The technology makes it possible to achieve different filling of the surface with zinc oxide nanorods. The nanorods is partially fused into the polymer; the cross section of the nanorods is rather close to an elongated ellipse. It is shown that, with intense abrasion, no delamination of the nanorods from the acrylic polymer is observed. The zinc oxide nanorods abrades together with the acrylic polymer. Zinc oxide nanorods luminesces with the wavelength most preferable for the process of photosynthesis in higher plants. It was shown that plants grown under the obtained material grow faster and gain biomass faster than the control group. In addition, it was found that on surfaces containing zinc oxide nanorods, a more intense formation of such reactive oxygen species as hydrogen peroxide and hydroxyl radical is observed. Intensive formation of long-lived, active forms of the protein is observed on the zinc oxide coating. The formation of 8-oxoguanine in DNA in vitro on a zinc oxide coating was shown using ELISA method. It was found that the multiplication of microorganisms on the developed material is significantly hampered. At the same time, eukaryotic cells of animals grow and develop without hindrance. Thus, the material we have obtained can be used in photonics (photoconversion material for greenhouses, housings for LEDs), and it is also an affordable and non-toxic nanomaterial for creating antibacterial coatings.

## 1. Introduction

Currently, LED technology has become a part of our life [1]. LED sources consist of three main elements: a semiconductor crystal, a transparent body (reflector or lens), and conductors [2]. In this case, only the diode body interacts with the outside world. The diode body must be transparent, usually made of acrylic polymers or polystyrene [3]. It is known that under conditions of high temperature and humidity, for example, in greenhouses, various forms of life can comfortably develop on the surface of these polymers [4]. In this regard, an important aspect of new materials is the presence of bactericidal properties [5]. Many metal oxides, such as ZnO, TiO_2_, MgO, Fe_2_O_3_, and others, have significant bactericidal properties [6,7,8]. When these oxides are applied to surfaces, they retain high thermal and chemical stability and often have high mechanical strength. A thin coating of ZnO is transparent in the visible range of the spectrum [9], which means that such a coating can be used very widely, from glazing greenhouses and buildings to creating screens for gadgets. Zinc oxide nanorods are the most effective against microorganisms. Moreover, their antibacterial properties significantly depend on the microstructure [10]. It is possible to enhance the antibacterial properties by parallel spatial orientation of one-dimensional ZnO nanowires [11].

It should be noted that coatings based on ZnO and related compounds exhibit not only bactericidal properties but also have significant photocatalytic properties [12]. Antibacterial and photocatalytic properties are interrelated; more precisely, the antibacterial properties of ZnO increase when exposed to light. This is implemented as follows: (1) excitation of a semiconductor crystal with light; (2) the formation of electron-hole pairs; (3) generation of ROS on the surface of the material; (4) damage of biomolecules from ROS; (5) and bacteria death [13]. The main problem with antibacterial materials is the danger of these materials to the environment and human health. One of the trends in the development of antibacterial materials is the production of materials that affect the growth and development of bacteria but mostly do not affect the growth and development of mammalian cells. It should be noted that this problem is far from a successful solution [14]. 

Another important problem that thin coatings can take part in is the conversion of light from a semiconductor crystal. LEDs often provide a rather narrow spectral line, which is broadened by one or more fluorophores [15]. This technology is often used in commercial LED “white” light sources. It is known that plants most efficiently absorb light quanta in the spectral ranges of 400–490 nm and 630–710 nm [16]. In practice, LEDs with a corrected spectrum are used in greenhouses [17]; in addition, photoconversion materials are used to correct the spectrum of the sun [18]. It should be noted that nanosized fluorophores are intensively used in various fields of human activity [19,20,21,22]. It was previously reported that thin coatings consisting of ZnO-based nanorods are capable of intense luminescence when illuminated with ultraviolet radiation [23]. This phenomenon largely depends on the location and orientation of the nanorods. At present, no technologies have been developed to achieve a comprehensive orientation of nanorods on the surface of materials [24]. 

For the manufacture of coatings from metal oxides, various technologies are used, such as hydrothermal synthesis [25]; sol-gel synthesis [26]; polymer-salt synthesis [10]; PVD methods, including magnetron sputtering [27]; and others. For practical application, it is important that the method of obtaining bactericidal coatings provides high productivity and is simple [28]. The first three methods are technologically simple and inexpensive; however, these methods do not allow one to obtain both their zinc oxide nanorods and to influence the orientation of nanoscale objects on the substrate. Apart from sputtering, other physical vapor deposition (PVD) methods, such as arc evaporation, do not guarantee the growth of the necessarily ordered structures of zinc oxide and, as a rule, have too high deposition rates. On the other hand, presence of a substantial ionic flux to the sample surface, which distinguishes magnetron sputter devices from evaporators, allows achieving the growth of the required structures. In this work, we propose a method for producing oriented nanoscale wires from zinc oxide. The method used is based on magnetron sputtering on acrylic plastic under established boundary conditions. The magnetron’s magnetic system is specially arranged to obtain maximum ion flux in the direction of the substrate. It is shown that the resulting coatings have a luminescence stimulating the growth and development of plants. In addition, the ZnO-based coatings show significant antibacterial properties while exerting a minimal effect on the viability of mammalian cells.

## 2. Materials and Methods

### 2.1. Magnetron Sputter Deposition of Zinc Oxide on Acrylic Polymer

**Figure 1:**
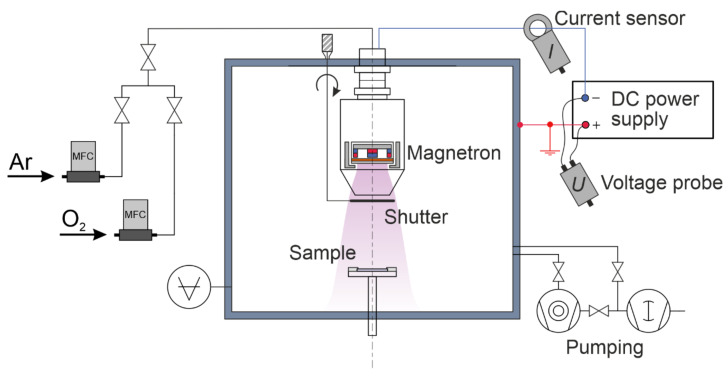
Schematic of a magnetron deposition setup.

ZnO coatings were deposited on plastic samples in a dedicated ion-plasma processing setup with a Magneto series magnetron (Pinch, LLC, Moscow, Russia). The setup diagram is shown in Figure 1. A planar circular magnetron with a target 76.2 mm in diameter and 2-mm thick was used to prepare the samples. The magnetron was capable of changing the magnetic field configuration by varying the positions of the magnets. The target was made of metallic zinc grade C0, purity 99.975% (Girmet, LLC, Moscow, Russia). Heat removal from the target to a water-cooled copper cathode was provided with KPT-8 thermal paste. The magnetic field of the magnetron was tuned to optimize the ion current value on the substrate. In the present experiments, we used the magnetic configuration that maximized the ion assistance of the film growth on the sample surface. Square, acrylic, 2-mm thick plates were used as substrates. Before being placed in a vacuum chamber, the surface of the samples was not subjected to any cleaning procedures. During deposition, each sample was located at a distance of 100 mm from the target surface on a special stage.

**Figure 2:**
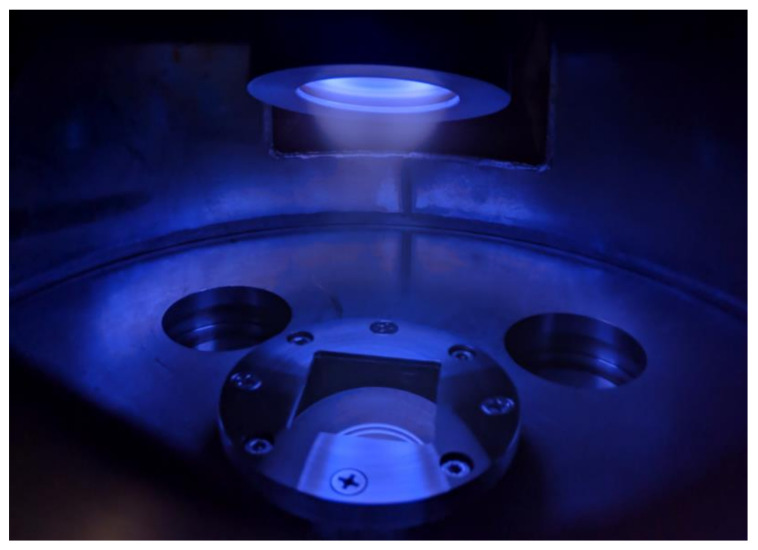
Photograph of the magnetron and the sample stage during the coating process.

The residual background pressure in the vacuum chamber during evacuation by a turbomolecular pump was less than 10^−4^ Pa. All coatings were obtained in the reactive magnetron mode by Zn target sputtering in Ar with the addition of O_2_ in a 1:1 gas flow rate ratio. This ratio was set by an automated gas-injection system based on Bronkhorst El-Flow flow controllers. In our case, the total pressure during deposition was 0.5 Pa, and the total flow rate of the gas mixture was 1.8 L/h or 30 sccm. A direct current (DC) magnetron discharge with a fixed power of 100 W was created in this gas mixture once a sufficient voltage was applied to the cathode. Typical discharge voltage was 340–360 V and discharge current 0.28–0.29 A, as provided by the APEL-M series power supply (Applied Electronics, LLC, Moscow, Russia). During the first 2 min after switching on, the magnetron was shielded with a special shutter to ensure the stabilization of the discharge parameters and increase the reproducibility of the results of coating deposition on the samples. Then, the shutter was opened, and the deposition process was started. The substrate temperature during deposition did not exceed 60 °C. The deposition process in the facility is illustrated in Figure 2. 

### 2.2. Manufacturing of Acrylic Polymer

Acrylic polymer in the form of rectangular sheets was obtained using an extruder. The raw material was granular polymethyl methacrylate. The pellets melt in the extruder to form a viscous liquid. The extruder itself is made in the form of a cylinder, inside which there is a spiral screw that mixes the molten granules and turns them into a homogeneous viscous mass. In addition, the screw creates excess pressure in the area of the die slot. After exiting the die, the acrylic polymer is pulled between the rotating rolls. This allows us to obtain a uniform thickness of the workpiece over the entire surface.

### 2.3. Characterization of the Surface Morphology of a Polymer with Zinc Oxide Nanorods

Photographs of the products were taken using an S8+ camera (Samsung, Suwon, Korea). A Vega3 scanning electron microscope (Tescan, Brno, Czech) was used. Nanoscale surface topology was explored using atomic force microscope, Nanopics 2000 (Seiko, Tokyo, Japan), and high-resolution laser modulation interference microscope MIM 321 (Amphora Lab, Moscow, Russia). The photoconversion properties of the AP-ZnO material were investigated using a fluorimeter 8300 (Jasco, Tokyo, Japan) in the mapping mode.

### 2.4. Cultivation of Plants

The effect of light modulated by photoconversion films on the morphometric parameters of plants was estimated in a climatic room. The plants were grown in soil. We tested representatives of important crops: cucumber (*Cucumis sativus*) and chili pepper (*Capsicum annuum*). The main morphometric indicator assessed was area of leaves. Image processing and calculations were made using original GreenImage V.1.0 software developed by our team. The algorithm of the program and the download link were published earlier [18].

### 2.5. Bacteriostatic Activity Assay

Experiments in a cultural environment. Gram-negative bacteria Escherichia coli were cultured [29]. Using aseptic techniques, we carefully transferred a 5-mL aliquot of LB broth into a sterile, lidded glass culture tube. Using a sterile applicator stick, one well-isolated colony was transferred from the solid medium plate to the culture tube. Then, the colony was resuspended in a glass culture tube. To determine the concentration of bacteria, a spectrophotometric study was carried out. The optical density of the resulting medium was determined using a drop spectrophotometer UV5Nano Excellence (Mettler Toledo, Columbus, OH, USA). For analysis, 10 μL of the medium containing the bacteria was irreversibly taken. After determining the concentration of bacteria, the resulting concentrated medium containing bacteria was diluted in a larger volume. For the experiments, films of a composite material with a thickness of 700–900 μm and a size of 10–15 mm were made. The film was sterilized by soaking three times in ethyl alcohol for 30 min. After that, the film was put on a round, sterile hoop. A nutrient medium with bacteria was poured into the hoop, and the top of the hoop was sealed with a piece of glass slide. The resulting structure was placed in an ES-20 incubator shaker (Biosan, Riga, Latvia) (37 °C, approximately 150 rpm). During incubation, the concentration of bacteria was estimated using microscopy and an algorithm developed by us for determining optically dense objects in the frame. At the end of the experiment, the structures were disassembled, and the concentration of bacteria was estimated again using a drop spectrometer. 

### 2.6. Cell Culture

**Figure 3:**
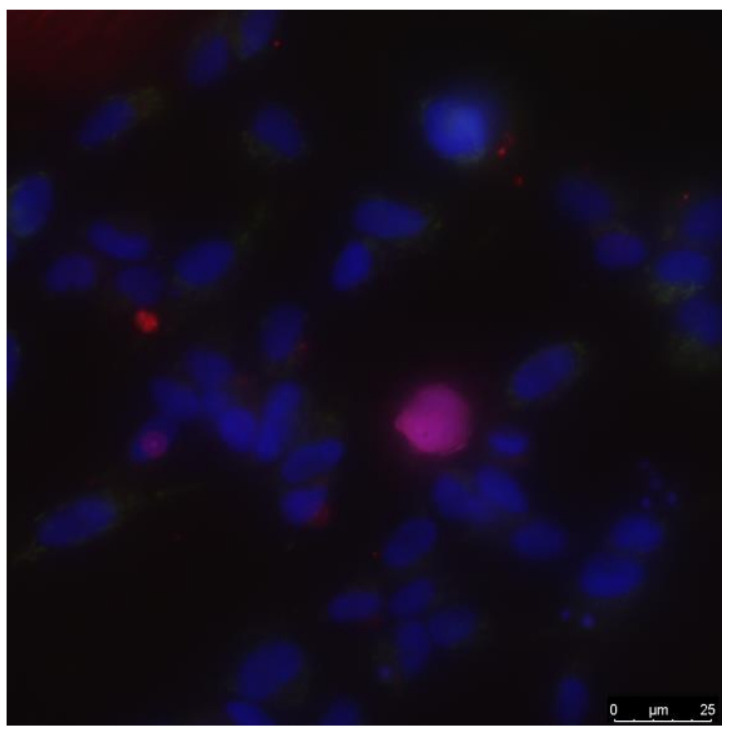
A typical photograph of a cell culture taken using a fluorescence microscope.

Biocompatibility studies were performed using standard in-vitro test systems. The SH-SY5Y human neuroblastoma cell culture was used as standard cell models. The cells (Thermo Fisher Scientific, Waltham, MA, USA) were grown in a DMEM medium (Biolot, Moscow, Russia) supplemented with 10% fetal calf serum (Gibco, Waltham, MA, USA), 30 μg/mL gentamicin at 37 °C, and 5% carbon dioxide in a CO_2_ incubator (Binder, Tuttlingen, Germany). Then, cells with a concentration of 10^4^ cells/cm^2^ were inoculated on the surface of material samples in a volume of 3 mL per dish. Cells were cultured on the samples for 72 h. Cells growing on the samples’ surface were stained with fluorescent dyes, 2 μg/mL Hoechst 33342 (Sigma, Saint-Louis, MO, USA) and 2 μg/mL propidium iodide (Sigma, Saint-Louis, MO, USA), to determine the number of live and dead cells, respectively (Figure 3). Hoechst 33342 stains all cells (live and dead, Sigma, Saint-Louis, MO, USA). The propidium iodide dye penetrates extremely slowly into live cells; therefore, during a short incubation time (about 10 min), it stains only cells with a damaged plasma membrane. The plasma membrane with breaks leading to dye penetration was one of the main criteria for a cell to be dead. Thus, Hoechst 33342 stains both live and dead cells, while propidium iodide only stains dead cells. Microscopic assay of the samples was carried out with an imaging system based on Leica DMI6000 (Leica, Berlin, Germany). At least 500 cells were counted on the surface of each sample for analysis [30]. The mitotic index of cells in the logarithmic growth phase (3 days after seeding) was used to analyze cell proliferation. The number of cells in a state of mitosis was determined using fluorescence microscopy using in-vitro staining with the Hoechst 33342 fluorescent dye (Sigma, Saint-Louis, MO, USA) [31]. The density of the culture was assessed using the approaches developed earlier [32].

### 2.7. Determination of the Concentration of Reactive Oxygen Species

To determine the concentration of hydrogen peroxide, we used the method of enhanced luminescence in the luminol-p-iodophenol-horseradish peroxidase system [33]. Measures were carried out with ultrasensitive chemiluminometer Biotox-7A-USE (ANO Engineering Center-Ecology, Moscow, Russia). The sensitivity of the method makes it possible to determine H_2_O_2_ at a concentration of <1 nM [34]. Evaluation of OH-radicals concentration was carried out using the reaction with coumarin-3-carboxylic acid (CCA), which led to the hydroxylation of CCA to 7-hydroxycoumarin-3-carboxylic acid (7-OH-CCA). The 7-OH-CCA is a convenient fluorescent probe for determining the formation of these radicals. The fluorescence of 7-OH-CCA (the product of the reaction of CCA with a hydroxyl radical) was measured with spectrofluorimeter 8300 (JASCO, Tokyo, Japan) with λ_ex_ = 400 nm, λ_em_ = 450 nm. Calibration was performed using commercial 7-OH-KKK. Methodical details have been described in detail earlier [35].

### 2.8. Determination of Biomacromolecules Damages

A non-competitive enzyme-linked immunosorbent assay (ELISA) using monoclonal antibodies specific to 8-oxoguanin (anti-8-OG antibodies) was used to quantitatively measure 8-oxoguanine in DNA. The optical density of samples was measured with a plate photometer FX (Titertek Multiscan, Vantaa, Finland) at λ = 405 nm. The method was described in more detail earlier [36]. Long-lived reactive protein species concentration were measured using a chemiluminescence method. The method was described in more detail earlier [37].

## 3. Results

### 3.1. Material Surface Morphology

**Figure 4:**
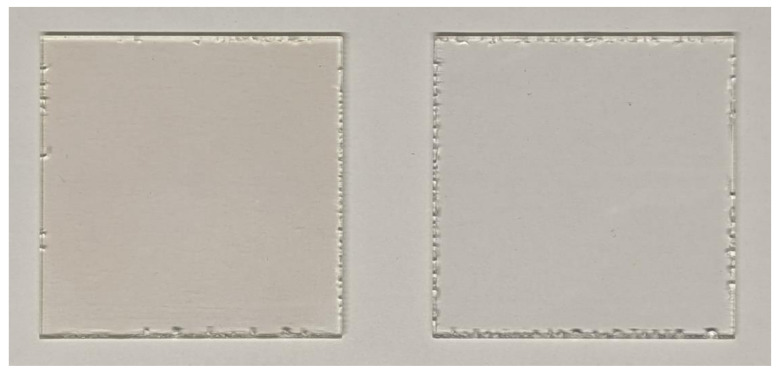
Photographs of samples with ZnO coating (**left**) and without it (**right**).

The nanoscale zinc oxide layer deposited on an acrylic polymer is visible on the surface of the material (AP-ZnO) only in bright light and only at a certain angle. Figure 4 shows photographs of samples with and without ZnO coating. Interestingly, with intense abrasion, no delamination of the zinc oxide layer from the acrylic polymer is observed. Zinc oxide abrades together with the acrylic polymer.

**Figure 5:**
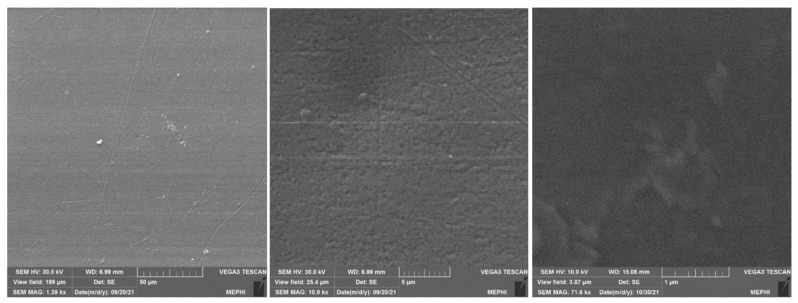
SEM images of a ZnO coating surface with a deposited Ag layer at different scales.

The deposited ZnO coatings had too low electrical conductivity and could not be directly diagnosed in a scanning electron microscope. To obtain images of the surface, an additional 40-nm thick Ag coating was deposited. Figure 5 shows SEM (Tescan Vega 3, Brno, Czech) images of the surface at various scales. It is shown that the surface is flat on a scale of tens of micrometers. On a micrometer scale, the samples exhibit longitudinal and transverse grooves. One should keep in mind that we had to deposit a layer of silver on zinc oxide. How does this layer affect the surface morphology of the object? Is the surface morphology of zinc oxide and silver different? It is obvious that it is rather problematic to find an answer to these questions with the help of scanning electron microscopy.

**Figure 6:**
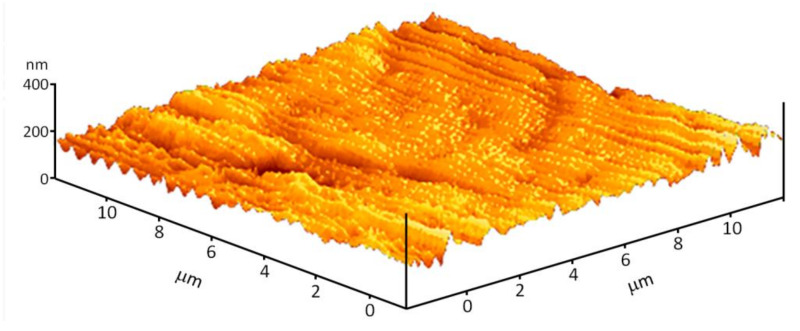
Reconstruction of the surface of the AP-ZnO material using atomic force microscopy.

The morphology of the material surface was investigated using an atomic force microscope (Figure 6). It is shown that the surface of the material is generally homogeneous. Longitudinal and transverse grooves are observed on the surface. In general, the direction of the grooves is ordered. The average groove depth is about 50–100 nm. An atomic force microscope is able to obtain information about surface topology but cannot determine the mutual distribution of the materials, acrylic polymer and zinc oxide.

**Figure 7:**
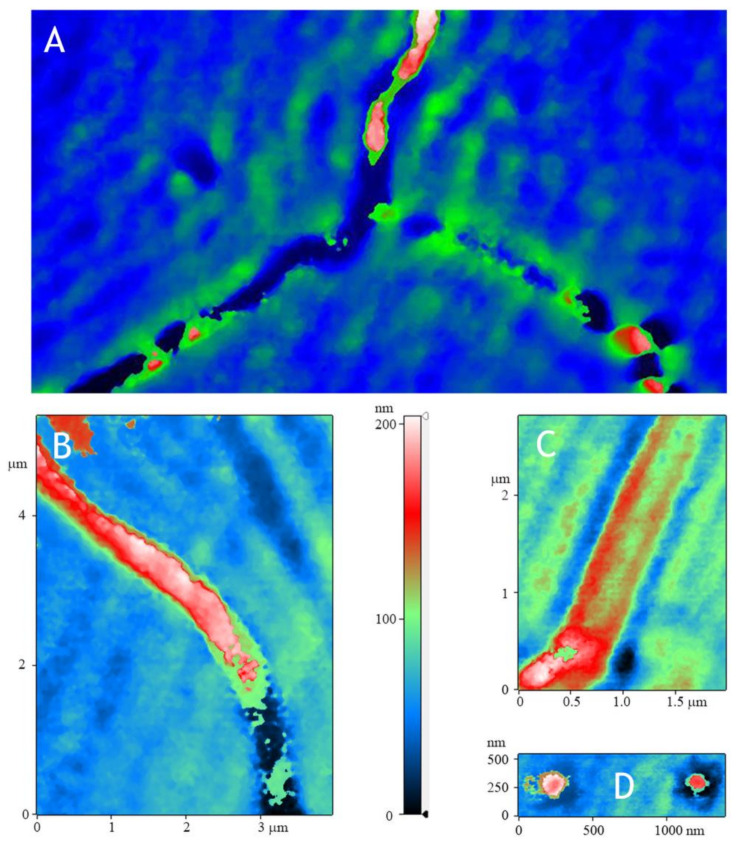
Reconstruction of the surface of the AP-ZnO material using modulation interference microscopy. (**A**) General view. (**B**,**C**) Maps with characteristic zinc oxide fusions. (**D**) Map with a single point of growth of zinc oxide.

It is known that acrylic and zinc oxide differ significantly in their optical properties. In this regard, we used a modulation-interference microscope. This microscope allows us to build 2D maps showing how the phase incursion changes at a particular point in space. In the case of optically opaque objects, the microscope works as a high-precision profilometer. Using modulation interference microscopy, it was shown that during deposition, zinc oxide is distributed on the plastic in the form of oriented stripes (Figure 7A, green). Additionally, after spraying on acrylic, the formation of deep grooves is observed (in the photo, there are three grooves converging in the center) in which the growth centers of zinc oxide are located. Interestingly, grooves form in acrylic but are not always filled with zinc oxide. At higher magnification, it can be seen that, often, such grooves are adjacent to less deep grooves running both along and across (Figure 7B,C). The size of such grooves varies from 150 to 500 nm. Zinc oxide growth centers in the form of droplets 150–200 nm wide are also found on the polymer (Figure 7D). In general, two types of polymers have been investigated. In the first type of polymers, approximately 25% of the area is covered with zinc oxide. The second type of polymer is approximately half coated with zinc oxide. The above observations are valid for both types of polymers, both with a coverage of 25% of the surface and with a coverage of 50% of the surface.

**Figure 8:**
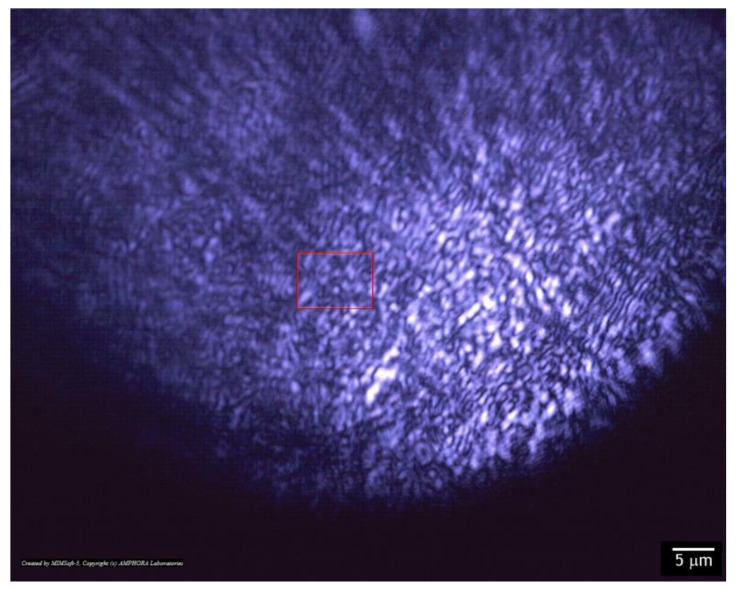
Photograph of AP-ZnO material obtained using a laser microscope.

The data from the modulation interference microscope are confirmed by laser microscopy in transmitted light mode (Figure 8). It is shown that the surface of the material is dotted with grooves extending in different directions. The size of the grooves obtained by laser microscopy and by means of modulation interference microscopy is correlated. 

**Figure 9:**
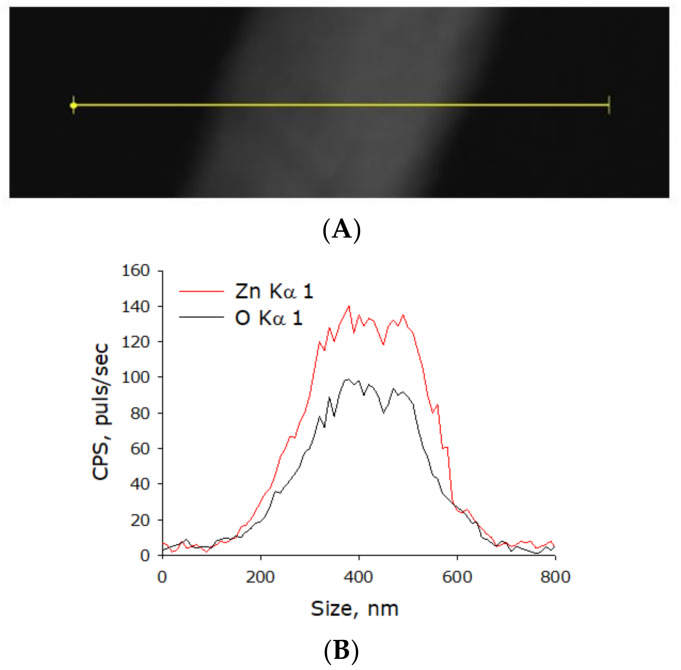
Elemental analysis of zinc oxide nanorods. (**A**) TEM image of group of zinc oxide nanorods; analysis section is indicated by line. (**B**) Nanorods profile by Zn Kα1 and O Kα1.

Separately, elemental analysis was done (Figure 9). The chemical composition of the nanorods was confirmed.

### 3.2. Photoconversion Properties of the Material

**Figure 10:**
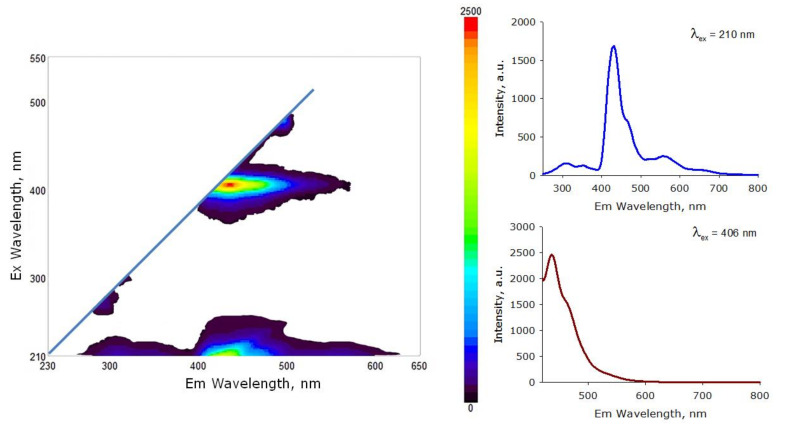
Photoconversion properties of AP-ZnO material. On the left is a 2D graph characterizing the fluorescence intensity of the material at all investigated wavelengths. On the right are the material fluorescence spectra upon excitation at 210 nm (**top**) and 406 nm (**bottom**).

The photoconversion properties of the AP-ZnO material were investigated (Figure 10). Using a fluorometer equipped with shutter, the fluorescence of the AP-ZnO material was investigated at excitation from 210 to 520 nm with a step of 1 nm. It was shown that the strongest fluorescence is observed upon excitation at wavelengths close to 210 and 406 nm. Weak luminescence directly behind the shutter line can be seen with excitation in the 250–300 and 430–500 nm wavelength ranges. When excited at 210 nm, the material luminesces over the entire wavelength range from 280 to 700 nm. A pronounced maximum of the luminescence intensity is observed at 430 nm. Local maxima are observed at 300 and 560 nm. When excited at 406 nm, the material has maximum luminescence at 435 nm. The spectral range in which the luminescence of the material is observed is from 420 to 550 nm.

**Figure 11:**
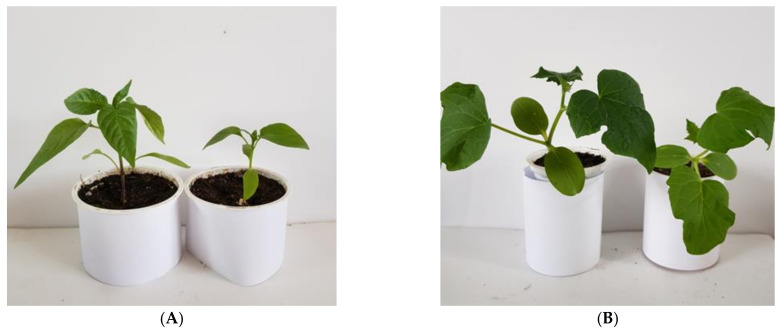
Effect of photoconversion polymer film on plant leaf size. Representative photographs of plants grown under AP-ZnO photoconversion material (**left in pair**) and acrylic polymer (**right in pair**). (**A**) Cucumber (*Cucumis sativus*). (**B**) Chili pepper (*Capsicum annuum*).

The AP-ZnO photoconversion material has a luminescence maximum of 430 nm. It is known that plants most efficiently absorb light quanta in the spectral ranges of 400–490 nm and 630–710 nm. We assumed that the material we made could be used as a photoconversion coating for greenhouses. It has been shown experimentally that the photoconversion material of the AP-ZnO material increases the growth rate of cucumber and pepper plants (Figure 11). The highest growth rate is observed in pepper plants. It has been shown that the leaf area of pepper plants grown under a photoconversion coating by the end of three weeks of vegetation is 35–40% larger than in the control group. Cucumber leaves amassed only 25–30% more leaf area than control. 

### 3.3. Biosafety

**Figure 12:**
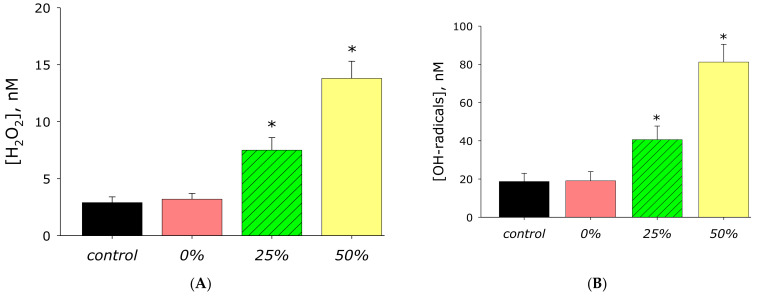
Effect of material AP-ZnO on the reactive oxygen species generation. (**A**) Generation of hydrogen peroxide (2 h, 40 °C). (**B**) Generation of hydroxyl radicals (2 h, 80 °C). * indicate a significant difference at 5% level in comparison with the control (*p* < 0.05). Data are presented as mean values and standard errors.

The effect of zinc oxide nanorods on the generation of reactive oxygen species (ROS) in aqueous solutions was studied. It was shown that acrylic does not significantly affect the generation of ROS (Figure 12). At the same time, the AP-ZnO material with a coverage of 25% of the area with zinc oxide more than doubles the yield of hydrogen peroxide. AP-ZnO coated with 50% of the area with zinc oxide increases the yield of hydrogen peroxide by almost five times. AP-ZnO material coated with 25% of the area with zinc oxide more than doubles the yield of hydroxyl radicals. At the same time, the AP-ZnO material coated with 50% of the area with zinc oxide increases the production of hydroxyl radicals by more than 4 times.

**Figure 13:**
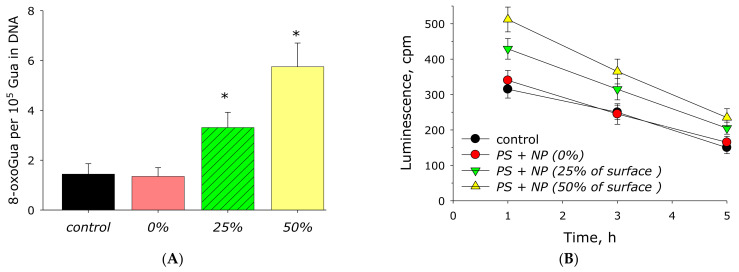
Effect of material AP-ZnO on the generation on biomacromolecules damage. (**A**) Generation of 8-oxoguanosine in DNA in vitro (2 h, 45 °C). (**B**) Formation of long-lived reactive protein species (2 h, 40 °C). * indicates a significant difference at 5% level in comparison with the control (*p* < 0.05). Data are presented as mean values and standard errors.

It is known that excessive generation of ROS is associated with damage to biomacromolecules in living cells and biological fluids. The effect of the AP-ZnO material on the formation of such a key marker of oxidative stress as 8-oxoguanine in DNA in vitro was studied (Figure 13A). It was found that acrylic does not significantly affect the formation of 8-oxoguanine in DNA in vitro. The rate of 8-oxoguanine formation in DNA significantly increases with the appearance of zinc oxide in the polymer. The AP-ZnO material coated with 25% of the area with zinc oxide more than doubles the yield of 8-oxoguanine in DNA. AP-ZnO coated with 50% of the area with zinc oxide increases the yield of 8-oxoguanine by almost four times.

The effect of AP-ZnO material on the formation of long-lived active forms of proteins was studied (Figure 13B). It has been shown that acrylic does not affect both the formation and the rate of decomposition of long-lived, active forms of proteins. The sputtering of zinc oxide onto the polymer leads to a significant increase in the rate of generation of long-lived, active forms of proteins. An increase in speed of 35% occurs on a material coated 25% by zinc oxide. AP-ZnO coated on 50% of the area with zinc oxide increases the yield of long-lived, active forms of proteins by more than 60%. At the same time, zinc oxide coating practically does not affect the average half-life of long-lived, active forms of proteins. The half-life of long-lived, active forms of proteins is about 4–5 h in all experimental groups.

**Figure 14:**
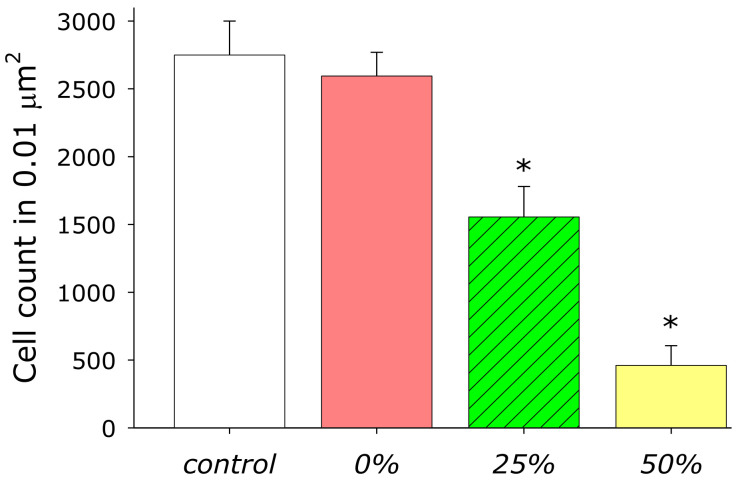
Influence of material AP-ZnO on *E. coli* growth and development. * indicates a significant difference at 5% level in comparison with the control (*p* < 0.05). Data are presented as mean values and standard errors.

The influence of materials with zinc oxide sputtering on the growth and development of *E. coli* bacteria was investigated (Figure 14). It was shown that the addition of acrylic does not significantly affect the growth and development of bacteria. When AP-ZnO material coated on 25% of the area with zinc oxide is added to the culture, a decrease in cell concentration by about 45% is observed. When AP-ZnO material coated on 50% of the area with zinc oxide is added to the culture, a decrease in cell concentration by more than 80% is observed.

**Figure 15:**
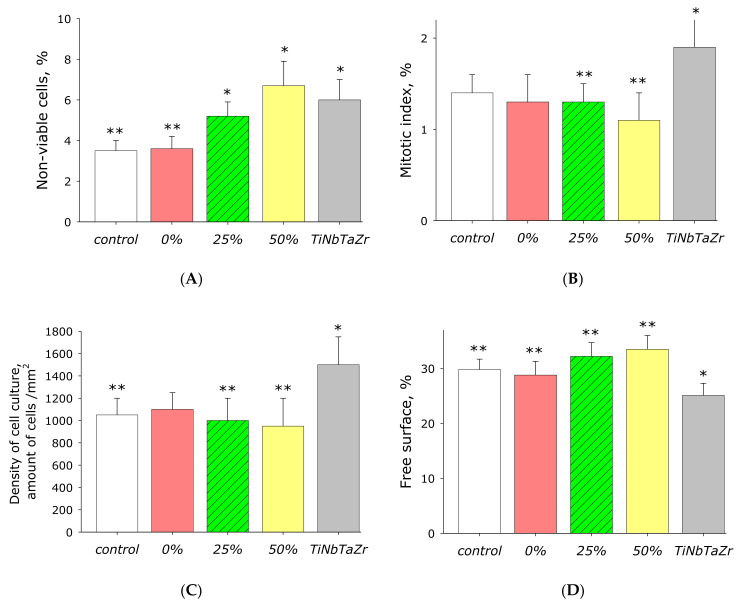
The effect of material AP-ZnO on the main characteristics of the growth and development of cell cultures. (**A**) Effect of material AP-ZnO on cell viability. (**B**) Effect of material AP-ZnO on the mitotic index of the cell. (**C**) Effect of material AP-ZnO on cell culture density. (**D**) Effect of material AP-ZnO on the rate of colonization of the available area. TiNbTaZr is a medical alloy based on the elements titanium, niobium, tantalum, and zirconium. * indicates a significant difference at 5% level in comparison with the control (*p* < 0.05). ** indicates a significant difference at 5% level in comparison with the group TiNbTaZr (*p* < 0.05).

The effect of AP-ZnO material on the viability of mammalian cells was studied (Figure 15A). The number of non-viable cells grown on control substrates did not exceed 4%. Approximately the same number of non-viable cells was observed when grown on untreated acrylic. The medical alloy TiNbTaZr was used as a negative control. When using this alloy as a substrate, non-viable cells were observed almost 6%, almost 50% more than in the control. On AP-ZnO material coated on 25% of the area with zinc oxide, almost 50% more non-viable cells were observed compared to the control. On AP-ZnO material coated on 50% of the area with zinc oxide, almost 90% more non-viable cells were observed compared to the control. It should be noted that the proportion of non-viable cells on AP-ZnO materials did not differ statistically from the number of non-viable cells on the TiNbTaZr medical alloy. 

To determine the ability of cells to divide, the mitotic index of cells in the phase of logarithmic growth was calculated (Figure 15B). It was found that the mitotic index of the culture of cells growing on the surface of control samples is 1.4%. When TiNbTaZr is used as a substrate, the mitotic index is almost 2%. The mitotic index of cells on untreated acrylic on AP-ZnO materials coated on 25 and 50% of the area with zinc oxide did not differ from the control.

The density of the cell culture was determined after 72 h of culturing cells on the surface of the materials. The density of the cell culture grown under control conditions averaged about 1000 cells/mm^2^ (Figure 15C). The density of cells grown on TiNbTaZr is almost 1.5 times higher than in the control. The density of cells grown on AP-ZnO material is in the range of 950–1100 cells/mm^2^.

The surfaces of AP-ZnO materials have been shown to be suitable for cell attachment and spreading (Figure 15D). After 72 h of cultivation on the surface of all samples of materials, the cells did not form a continuous monolayer; however, some elements of the monolayer were observed. Cells on all materials occupy about three-quarters of the surface available for growth. Moreover, according to the degree of suitability, AP-ZnO materials are comparable to culture plastic; however, they are 15% worse than the surfaces of the TiNbTaZr medical alloy.

## 4. Discussion

In this work, using the additive technology, a thin, acrylic polymer with a nanosized ZnO coating was obtained. The coating was examined using scanning electron microscopy (Figure 5), atomic force microscopy (Figure 6), modulation interference microscopy (Figure 7), and laser microscopy (Figure 8). It was shown that the most adequate method for obtaining information about the coating is modulation interference microscopy. Using this method, it was possible to show that during deposition, zinc oxide is distributed on the plastic in the form of oriented stripes (Figure 7A). It looks like a flat nanorods fused into a polymer. This achievement is based on: (1) selection of polymer and (2) fine tuning of the installation for sputter deposition. The attachment of the zinc oxide nanorods to the polymer is extremely strong. Abrasion of such a wire will occur only with abrasion of the polymer. In principle, ZnO nanorods were obtained earlier [38]. However, in this work, the nanorods were obtained by magnetron sputter deposition onto the sample. In this work, we investigated two types of polymers (surface coverage by 25 and 50%). In principle, the method we propose makes it possible to achieve any filling of the polymer surface.

It was shown earlier that zinc oxide coatings and crystals are capable of intense luminescence [39]. The coating we applied also exhibits powerful luminescence when excited at wavelengths close to 210 and 406 nm (Figure 10). When the coating is excited in the ultraviolet range, the material luminesces in the entire wavelength range from 280 to 700 nm, with a pronounced maximum luminescence intensity at 430 nm. It is known that plants photosynthesize most efficiently in the spectral ranges of 400–490 nm and 630–710 nm [40]; that is, the AP-ZnO material can potentially increase plant productivity. Currently, a large number of photoconversion materials are known [41,42,43,44,45,46]. At the same time, not many materials can boast of an increase in biomass productivity by 30%. Usually, the maximum luminescence of zinc oxide is near 380–390 nm [47]. It is known that the geometry and morphology of zinc oxide crystals mainly affect the luminescence intensity [48]. In this case, the wavelength at which photoluminescence is observed largely depends on the properties of the substrate on which zinc oxide is deposited [49,50]. The luminescence of zinc oxide can even shift towards the yellow region of the spectrum [51].

It is known that generation of reactive oxygen species (ROS) is observed on the surface of zinc oxide coatings in in-vitro systems [52]. It is known that an increase in the intracellular concentration of ROS often leads to the development of oxidative stress [53] associated with lipid peroxidation [54], oxidative modification of proteins [55], and nucleic acids [56]. Damage to biological molecules is associated with processes, such as mutagenesis, carcinogenesis, teratogenesis, aging, etc. [57]. Intense ROS generation is potentially dangerous for all living systems [58]. This is especially true of physiological conditions associated with the development of diseases associated with oxidative stress [59]. In the presence of deposited ZnO, intense generation of ROS was observed (Figure 12). Moreover, the intensity of ROS generation is proportional to the degree of filling the material surface with zinc oxide. A similar behavior was observed in a large number of other systems in the presence of variable valence cations [60].

It is known that ROS formed under the action of external factors or endogenously are capable of damaging biological molecules and supramolecular formations [61,62]. The effect of the AP-ZnO material on the generation of such damage to proteins as long-lived, active forms of proteins was studied. Usually, long-lived, active forms of proteins are understood to mean long-lived protein radicals and protein hydroperoxides. It is known that long-lived, active forms of proteins can cause damage to nucleic acids [63]. In addition, they are one of the reasons for the prolongation of oxidative stress [64]. We have shown that the coating based on ZnO leads to the intensive formation of long-lived, active forms of proteins (Figure 13). It has also been shown that the AP-ZnO material leads to the formation of 8-oxoguanine in DNA, a key biomarker of oxidative DNA damage. 8-oxoguanine has the properties of ambiguous coding and can lead to the formation of mismatched nucleotides with adenine, which in turn makes GC-TA transversion possible [65]. In mammals, there are at least four complex mechanisms for removing 8-oxoguanine from DNA and for preventing its incorporation into DNA [66]. The presence of such a number of duplicating mechanisms suggests that the cell perceives 8-oxoguanine as an extremely serious threat that must be quickly eliminated [67].

Zinc, as a chemical element, is a rather toxic compound [68]. Fundamentally, the antibacterial activity of the AP-ZnO material (Figure 14) can be explained by several reasons: disruption of the cell membrane [69], binding to proteins and DNA [70], formation of reactive oxygen species (ROS) [71], impaired amplification of bacterial DNA [70], and changes (more often, suppression) of expression in a wide range of genes [72]. Interestingly, with a pronounced antibacterial activity, AP-ZnO materials have almost no effect on the growth, development, and ability to colonize the surfaces of eukaryotic cells (Figure 15). It was found that cells growing on the TiNbTaZn medical alloy and on the AP-ZnO material have similar growth and development indicators. It should be noted that alloys based on TiNbTaZn are quite modern and advanced alloys [73,74]. It was shown that alloys based on TiNbTaZn have better characteristics than the widely used alloy nitinol [75]. Thus, a technology has been developed for producing nanoscale-oriented zinc oxide nanorods on an acrylic polymer. The nanorods are partially fused into the polymer. It was found that the reproduction of microorganisms on the material AP-ZnO is significantly hampered. At the same time, eukaryotic cells of animals grow and develop without hindrance on the developed coating. The use of AP-ZnO as an affordable, cheap, and non-toxic nanomaterial for the creation of antibacterial coatings that prevent the appearance of biofilms seems to be very promising for a number of industries.

## Data Availability

No additional data available.
